# Governance Quality, Public Health, Education, and Innovation: Study for Novel Implications

**DOI:** 10.3389/fpubh.2022.940036

**Published:** 2022-07-07

**Authors:** Ning Wu

**Affiliations:** School of Journalism and Communication, Hubei University, Wuhan, China

**Keywords:** malaria incidence, educational expenditure, technological innovation, human capital, research and development, government health expenditure, quantile regression

## Abstract

Pandemic or worldwide disease is the greatest issue of all time that not only affects human health but also influences the economic, educational, and other activities of the countries, since malaria is among the leading health disease that disrupts the economic system of the country. Therefore, this study aimed to analyze whether educational expenditure and technological innovation influence malarial incidence in emerging economies. This study also examined the role of government effectiveness, government health expenditure, gross domestic growth, human capital, and research and development during the period 2000–2018. Employing panel data approaches, including the slope heterogeneity and cross-sectional dependence, the second-generation unit root test reveals the stationarity of all variables. The study also validated the existence of a long-run relationship between the variables. Based on the asymmetrical distribution properties, this study employed the quantile regression approach. The empirical results asserted that education and technological innovation significantly reduce malarial incidents in the panel economies. Also, government effectiveness, research and development, and human capital adversely affect incidences of malaria. In contrast, gross domestic product is the only factor found that increases malarial incidents during the selected period. Based on the empirical results, this study suggested policy measures that could benefit the governors, policymakers, and scholars.

## Introduction

**Figure 1:**
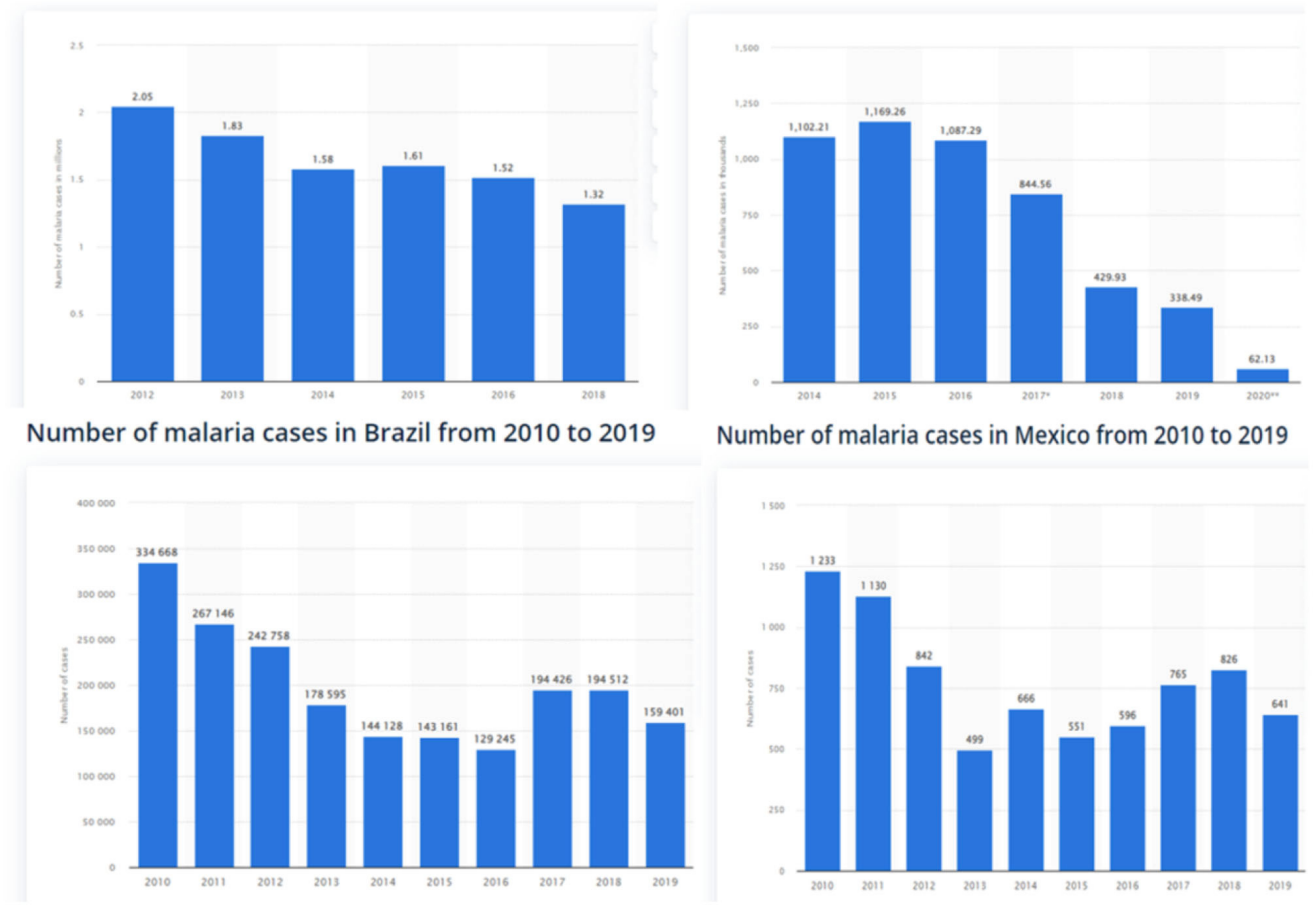
Number of malaria cases per year in Indonesia, India, Brazil, and Mexico Source: Statista 2022.

Malaria is an ancient life-threatening parasitic disease that is instigated by an *Anopheles* mosquito bite. According to the “United Nations children's fund,” it is the third major child killer in the world after pneumonia and diarrhea^1^. It is a major health concern that affects almost 350 to 500 million lives and causes one million deaths worldwide. In the year 2020, approximately 241 million cases of malaria were reported around the world, while in 2019, almost half a million people died globally (1), though, the past decades were considered fruitful in preventing and controlling malaria with ~60% reduction in disease spread according to the World Malaria Report (2). For this reason, WHO has initiated certain programs and strategies for combating malaria in malarial endemic economies, but malaria is a reemerging disease (or plague) that mostly occurs in tropical poor areas and nation-states. In the emerging economies such as Indonesia, India, Brazil, and Mexico, 1.32 million (2018), 62,130 (2020), 159,401 (2019), and 641 (2019) cases of Malaria were reported, respectively, which are signified in Figure 1. A total of six deaths have been reported in China in the year 2020 and 19 deaths have been testified in the year 2019^2^.

Several climatic and health factors that cause malarial incidents, attributable to the Management of Malaria (BBC), are poor hygiene, the presence of mosquitos, variability of climate and extremely high temperatures, increasing humidity levels, etc. (3). In the present study, various other macros (country level) factors have been discussed that cause an increase or decrease in malarial incidents which are never being considered for research purposes simultaneously. The current study aimed to scrutinize those factors. In essence, it is commonly distinguished as the improvement in health expenses, and economic growth (GDP) has a significant role in the reduction in (any) disease spread. As well, for successful diagnostic and treatment, prevention, and control of diseases tech advancement, education, and R&D have a noteworthy role. Technological innovation is essential for advancement and is joined at the hip of our daily lives. It helps in the early detection of diseases, and sometimes, the hidden causes and factors become visible and apparent that aid in disease cure. Research and development are required for knowledge and discovering new ideas (treatments/cures) for the management of a disease. Educational awareness programs for coaching basic prevention and remedial measures support disease control because education prevents half of the disease by providing the public with a better understanding and responsiveness toward the disease (4), whereas healthy human capital and effective government along with the certain implementation of SOPs can play a vital part in effectively controlling the disease and pave a means for economic development. Effective government increases health expenses on illness treatment, healthcare facilities/ services, and mobilization of health resources for healthcare development that eventually bring improved health outcomes and a lesser number of cases. Studies of Njau et al. (4), Christaki (5), Lee and Jung (6), and Omri et al. (7) highlighted the significance of these factors in the malarial disease, but still, the relationship among the variables is uncertain or not appropriately evaluated. The increase in health expenses and economic growth reduces the disease incidents (8, 9). Similarly, do technological innovation, human capital, research and development, education, and government effectiveness besides GDP and health expenses do the same with incidents of malaria in E6 countries which is a reemerging endemic around the world?

The study has the following objectives. First, the study aims to determine the role of the effectiveness of government, education, research and development, and government health expenditure over the malarial incidents (per 1,000 populations at risk) in the first econometric model. Second, the purpose of the study was to examine the influence of human capital, technological innovation, and gross domestic product, besides government effectiveness, over malarial incidents (per 1,000 populations at risk) in the second model. To accomplish these objectives and following the study (10), the authors employed variables with the inclusion of other factors such as technological innovation, government effectiveness, human capital, education, and research and development to evaluate malarial incidences. Hence, for assessing the impact of various factors on incidents of malaria in emerging six countries, the association is investigated by causal analysis and panel data approaches. The two modifications (Models 1 and 2) are elaborated in the methodology section.

A limited number of studies in the literature witnessed the said connotation. The lack of existing literature and uncertain relationships on the effect of government effectiveness, human capital, education, technological innovation, and research and development on the incidence of Malaria led to this research. According to the available knowledge of authors, Wei et al. (10) is the only novel study that has consciously examined the relationship between government health expenses, human capital, and GDP on malarial incidents and cases in the case of emerging economies. Despite this fact, some studies have emphasized the role of economic growth and health expenses on malaria cases and mortality rates. Therefore, the motivation of the study is to evaluate the effect of a diverse range of variables on incidents of malaria in six emerging nations, which is a topic of concern for many researchers in the exiting era. Second, malarial infection is ancient but is still prevalent in large proportions of the world that is not only affecting the daily lives and health of the individuals but also has a significant impact on the socioeconomic conditions of the country, which is hampering economic progress and putting barriers in way of the sustainable development goals. Consequently, the study incentivizes those factors of malarial incidents. The findings support in the prevention and eradication of the disease in the coming future.

The study contributes to the prevailing literature in the succeeding ways. A few studies examined the impact of some variables on disease incidents (8, 11–14) and were focused on health and economic growth with mortality cases. First, the present study is involved in assessing the effect of human capital, technological innovation, education, GDP, health expenses, and government effectiveness on malarial incidents. Therefore, the study contributes by exploring the said connection which has not been conducted till now. The study findings will be beneficial in health policymaking in order to fight against malaria because socioeconomical, ethical, and health measures are necessary to eradicate this disease. Second, the study has conducted a panel study in six emerging economies of the world such as China, India, Brazil, Turkey, Mexico, and Indonesia from the period 2000 to 2018 that have not been focused formerly on the prevailing literature, which is a novel input academically for emerging nations. Supplementarily, the present study extends the debate on malarial infection incidents in the case of emerging economies by including numerous variables in two different modifications together with employing a panel econometric analysis for scrutinizing the causal associations among them, which is innovative research in the speculative and empirical literature.

The succeeding section documents the review of existing literature to elucidate the variable connections and linkages for research. Section Data and Methodology is about the data, model, methodology, and econometric approach description. Section Results and Discussion deals with results and discussions, and Section Conclusion and Implications describes the conclusion and policy implications.

## Literature Review

This section of the manuscript provides a comprehensive review of the available literature on the aspects and connections of the dependent variable, which is malarial incidents with the other explanatory variables. The scarceness of studies in the existing literature concerning the association between infectious disease or malarial incidences with other factors underneath is cited in some available shreds of literature evidence that tend to elucidate the linkage between the study variables.

### Gross Domestic Product

Gross domestic product has a substantial influence on eliminating malarial infection incidences. Few authors have briefed the relationship. McCarthy et al. (15) described an inverse association between GDP and malaria prevalence. Orem et al. (8) analyzed that, in Uganda, the increase in malarial incidents has negatively impacted the GDP of the country, i.e., increasing one unit incident reduces USD$0.00767 of GDP per year. However, an improvement in the gross domestic product of a country can indirectly reduce malarial incidents despite ignoring other factors (14). Wei et al. (10) estimated that the rise in GDP influences the malaria cases or incidents representing a negative relationship with malaria spread (incidents). Sarma et al. (16) demonstrated that an increase in economic outcomes (GDP) by almost 0.3% substantially reduced malarial incidents by 10 percent. GDP (per capita) and incidents of malaria have shown inverse associations in the study. In another study by Kinyondo et al. (17), gross domestic product and malarial mortality incidents have depicted unidirectional causality running from GDP to incidents rates. The empirical findings show a negative relationship between the variables.

### Health Expenditure

Expenditures on health are essential for the spread of a certain disease whether it is a pandemic or endemic. The connection between health expenses and malarial incidents has usually an inverse association. Oluwaseyi et al. (18) examined the association between public health expenses and health outcomes considering malaria, HIV, infant, and maternal mortality rates in Ghana and Nigeria. Both countries had low public health expenses; however, Nigeria showed a positive association and Ghana has a negative relationship with health outcomes. Omri et al. (7) found that increasing health and R&D expenses aid in improving health outcomes. Wei et al. (10) scrutinized the relationship between government health expenses, human capital, economic growth, and human health considering malarial incidents and cases in emerging economies. The empirical findings demonstrated that improvement in health expenditures helps in reducing health disasters such as malarial cases and incidents in emerging nations depicting negative and Granger causes of malaria cases in the country. Nwanosike-Dominic et al. (9) analyzed health expenses through government substantially improve health outcomes that will reduce infectious disease spread and cases in Nigeria.

### Government Effectiveness

Limited studies have explored the relationship. Besides, Sarpong and Bein (19) scrutinized governance effectiveness as a negative association with malarial incidences in non-oil producing countries while a substantial positive impact is found in oil-producing economies. Liang et al. (11) examined the association of government effectiveness with infectious disease mortality cases and found an inverse relationship. The increase in effectiveness number substantially reduces the mortality incidences. Lee and Jung (6) described that government effectiveness and legislation play a substantial role in disease spread and control.

### Human Capital

Malaria is spread in human hosts through mosquito bites. The increasing population or human capital tends to increase infectious diseases and incidents attributable to increasing vulnerability (20). Goenka and Liu (21) explored infectious disease and human capital significance. Dash et al. (22) demonstrated that people in poverty-ridden areas have more significant chances of catching infectious diseases which led to increasing incidents, whereas areas having more economic development with population density have lesser chances of infectious diseases. As *per se*, health is considered another measurement of human capital. Manuelli (23) suggested that investing in human capital can help in fighting against diseases like malaria and AIDS that become barricades to economic growth. Wei et al. (10) found a bidirectional causal association between human capital and malarial incidences that human capital Granger causes in occurrence in incidents of malaria and *vice versa*.

### Education

Due to the scarcity of studies related to education and malarial incidences, the following studies might help clarify the association. It is stated that education plays a significant role in preventing infectious diseases (24). Rahman and Kuddus (13) inspected the malaria transmission dynamics. They concluded that lack of awareness or education substantially affects an increase in malarial incidences and severity. According to the RBM Partnership to end malaria, a global platform for eradicating malaria, a significant reduction in malarial incidences because of educational awareness was realized (25). Njau et al. (4) examined the role of maternal education on malarial infections (childhood). The findings concluded that educational awareness decreases the malarial infection burden (incidences) not only in children but also in all age groups. Wang et al. (12) studied the impact of education on the behavior of students on infectious diseases. The findings concluded that health education deliberately helps in preventing infectious diseases. Castro-Sánchez et al. (26) also explored health literacy's influence and infectious diseases and found significant relations.

### Technological Innovation

Early detection of malaria is positively related to malaria elimination in living beings, which is possible through technological advancement (27). Technological innovation or product innovation for healthcare helps in detecting symptoms of diseases beforehand and aids in treatment and prevention. Further, it is also labeled as a risk reduction strategy. Increased interaction with innovative technology for health is beneficial in controlling a certain disease (28, 29). Technology is now inseparable from our day-to-day lives. According to a novel study by Huang, Brouqui, and Boudjema (30), technological innovation has a positive influence on infection control and reduces disease spread. In a review article, Christaki (5) emphasized different technological methods for preventing and controlling infectious diseases. Bhowmick et al. (31) highlighted that the utilization of mobile technology supports the eradication of malarial infection.

### Research and Development

According to WHO Research and development blueprint, R&D is a prerequisite for the effective and immediate development of vaccines and treatments of various infectious diseases that are resourceful in reducing disease spread and incidents (1). While stabilizing the economies, research and development are essential in order to make authentic vaccines to control infectious disease spread. Research and development highlight certain factors that would help in tackling emerging infectious diseases (32). Omri et al. (7) depicted that improving research and development helps in enhancing health outcomes (i.e., reduced mortality rates and diseases). Anser et al. (33) estimated that the rise and fall of research and development expenses and other elements led to an increase in infectious disease cases across the nation-states. Further, attributable to the National Academic Press (34) that R&D confronts the transmittable disease threats is emphasized.

The subsequent Table 1 represents a summary of empirical studies related to some study variables to elaborate on the associations between the model variables whereas the review of all related articles is as aforementioned.

**Table 1:** Summary of empirical literature review.

**Author (Year)**	**Country** **(period)**	**Variables**	**Methodology**	**Findings**
Orem et al. (8)	Uganda 1997 to 2003	GDP and MI	Double log econometric model	MI decreases GDP
Njau et al. (4)	Angola, Tanzania, Uganda 2006 to 2009	Education and MI	Two-stage cluster sampling (MIS)	Education decreases malarial incidences
Kinyondo et al. (17)	Tanzania Mainland 2004 to 2015	GDP and MI	Correlation and Granger causality	Unidirectional association from GDP to MI
Wang et al. (12)	China 2012 to 2013	Education and MI	Questionnaire Survey	Health educational awareness prevents diseases
Lee and Jung (6)	South Korea 2003 to 2017	GEF and MI	Qualitative meta-analysis	GEF significant change in infectious disease spread
Nwanosike-Dominic et al. (9)	Nigeria 1970 to 2013	GHE and MI	Regression analysis	Health expenses help in malaria reduction
Sarma et al. (16)	180 countries 2000 to 2017	GDP and MI	OLS, fixed effect models, 2SLS	GDP and MI are negatively associated
Zhao et al. (14)	18 countries 2011 to 2016	GDP and MI	Spatial and temporal distribution	Improving GDP decreases MI
Liang et al. (11)	169 countries' cross-sectional data	GEF and MI	Multiple regression analysis	Negative association
Sarpong and Bein (19)	Sub-Saharan countries: oil and non-oil producing (2005 to 2017)	GEF and MI	GMM	Positively associated in oil-producing countries and negative association in non-oil economies
Omri et al. (7)	Saudi Arabia 2000 to 2018	Health RandD expenditures and MI	DOLS	Health RandD expenditures aid in decreasing MI
Oluwaseyi et al. (18)	Ghana and Nigeria (2000 to 2018)	GHE and MI	Linear Regression	Improvement in GHE negatively impacts MI Nigeria and a positive in Ghana
Wei et al. (10)	Emerging seven Economies (2000Q1 to 2018Q4)	GHE, HC, GDP, MI/MC	Quantile regressions	GHE, HC, and GDP impact the health outcomes

## Data and Methodology

### Data and Model Construction

Following the objectives and the literature discussed above, a total of eight variables have been taken into consideration. Specifically, this study observed the occurrence or outbreak of fatal diseases and their implications in disturbing various economic, educational, and government policies. In this regard, this study opted to examine the influence of various economic and noneconomic indicators on malarial incidence (*MI*) as this fatal disease not only adversely affects human health but also influences economic and educational activities (10). Concerning the primarily focused variables, this study considered two variables, including the education expenditures (*EDU*) and the technological innovation (*TI*), since the *MI* could have a substantial influence on disturbing economic, educational, and technological activities. Therefore, it is important to analyze whether the improved level of *EDU* and/or *TI* influences *MI*. Besides, this study also considered a list of control variables and aims to identify their impact on *MI*. Specifically, the list consists of economic growth (*GDP*), government effectiveness (*GEF*), government health expenditure (*GHE*), research and development (*R&D*), and human capital (*HC*). Data for all the variables have been extracted from several sources, covering the period from 2000 to 2018, since the emerging economies are more at risk of the said fatal disease as these economies are more concerned about their economic stability and sustainable development. Therefore, this study covers a list of emerging economies, including China, India, Turkey, Brazil, Indonesia, and Mexico. Since data for several variables are not available for the seventh emerging nation, i.e., Russia, therefore, this study excludes the said country from empirical analysis for the time being. The specifications and units and the sources of the data for each variable are provided in Table 2.

**Table 2:** Variables, their specifications, units, and sources.

**Variable**	**Specification and unit**	**Data source**
MI	Incidence of Malaria per 1,000 population at risk	http://apps.who.int/ghodata/
EDU	Education Expenditure in Current US dollars	https://databank.world-bank.org/source/world-development-indicators
TI	Resident Application Patent Numbers	https://databank.world-bank.org/source/world-development-indicators
GDP	A monetary valuation of all completed services and products created in a specific period, measured in constant 2015 US$	https://databank.world-bank.org/source/world-development-indicators
GEF	Government Effectiveness in a Percentile Rank	www.govindicators.org
GHE	Domestic general government health expenditure as a Percent of GDP	http://apps.who.int/nha/database
R&D	Research and Development Expenditures as a Percent of GDP	https://databank.world-bank.org/source/world-development-indicators
HC	Refers to the economic worth of expertise, skills, and knowledge of a worker, Index	www.ggdc.net/pwt

Following the study of Wei et al. (10), this study adopted the following model:

**Model 1**


(1) $$\begin{array}{l} MI_{it}\;=\;\alpha_{1}+\beta_{1}GEF_{it}+\beta_{2}EDU_{it}+\beta_{3}R\;\&D_{it}+\beta_{4}GHE_{it}\\ \;+\;\varepsilon_{it} \end{array}$$

**Model 2**


(2) $$\begin{array}{r} MI_{it}=\alpha_{1}+\beta_{1}GEF_{it}+\beta_{2}HC_{it}+\beta_{3}TI_{it}+\beta_{4}GDP_{it}+\varepsilon_{it}, \end{array}$$

where Model 1 reveals that GEF, EDU, R&D, and GHE are collectively the function of MI, while Model 2 indicates that GEF, HC, TI, and GDP are collectively the function of MI. Besides, the α′*s* and β′*s* are the intercepts and slopes, respectively, whereas “*i*” and “*t*” represent cross sections and time period, respectively. Moreover, the random error of the model is presented *via* the vector ε.

### Estimation Techniques

Since this study deals with panel data estimation, therefore, it is important to examine panel data characteristics by employing diagnostic tests. In this sense, the current study examines the slope heterogeneity and the panel cross-sectional dependence test, which reveals the heterogenous characteristics of slopes and the existence of cross-sectional dependence. Therefore, this study uses the second-generation panel unit root test. As the variables considered are stationary, therefore, the current study tested for the long-run equilibrium relationship, which is valid in the case of the emerging economies. Besides, this study utilizes the data normality test that illustrates irregular data distribution across the selected time period. Therefore, it is crucial to utilize an appropriate estimator that could tackle the issue of data non-normality. Consequently, this study uses panel quantile regression to address the said issue.

#### Descriptive Statistics and Normality Check

The current study initiates the empirical analysis section by evaluating the descriptive statistics and normality estimates. Specifically, the mean, median, and range (maximum and minimum) values are evaluated that summarize the entire dataset. In addition, the standard deviation is also assessed which indicates the general volatility of a variable. Moreover, this study also tested the normality of each variable under consideration. In other words, the skewness and Kurtosis demonstrate the wideness and peak of a distribution. Particularly, both these measures range the value between −2 and +2 for skewness and between −7 and +7 for Kurtosis (35). On the contrary, this study also uses a comprehensive test for normality, i.e., the Jarque and Bera (36) normality test, that treats the skewness and excess Kurtosis simultaneously and proposed them to be equal to zero as a null hypothesis. The standard formulation of the said test is expressed as follows:


(3) $$\begin{array}{r} JB=\;\frac{N}{6}\left(S^{2}+\frac{{\left(K-3\right)}^{2}}{4}\right).\;, \end{array}$$

#### Slope Heterogeneity and Cross-Sectional Dependence

After industrialization, there was a significant expansion in worldwide trade and globalization despite the fact that a number of factors impact an economy's dependence on other countries. Specialization of one economy in certain goods or services attracts the attention of other states and countries that rely on these types of goods and services. The fundamental reason for this dependence is to achieve multiple cultural, social, financial, economic, technological, technical, and health-related goals and objectives defined by governments or states. Depending on such factors, the economy of one nation may display parallels or differences in some sectors relative to the economies of other nations leading to an econometric issue of slope heterogeneity and cross-sectional dependence. This study employs estimation techniques for panel data, including slope heterogeneity and cross-sectional dependence. If such issues of slope heterogeneity and cross-sectional dependency are ignored, the econometric analysis may provide ineffective findings (37). In light of this, the slope coefficient homogeneity (SCH) of Pesaran and Yamagata (38) and the Pesaran (39) cross-sectional dependence (CD) diagnostic tests are used to investigate these two-panel data problems. Regarding the SCH test, the standard equation for estimation may be stated as follows:


(4) $$\begin{array}{r} {\hat{\Delta }}_{SCH}=\;\sqrt{N{\left(2k\right)}^{-1}}\left(N^{-1}\text{Ś }-K\right).\;, \end{array}$$

In addition to SCH, this test explicitly analyzes the adjusted SCH, which may be computed using the formula below.


(5) $$\begin{array}{r} {\hat{\Delta }}_{ASCH}=\sqrt{N}\sqrt{\frac{T+1}{2K\left(T-K-1\right)}}\left(N^{-1}\text{Ś }-2K\right).\;, \end{array}$$

The SCH test assumes homogeneous slope coefficients as the null hypothesis, whereas the alternative hypothesis may only be accepted if the statistics are statistically significant.

Similarly, cross-sectional dependency cannot be ignored since it may lead to a skewed estimate in an econometric investigation (40). In this case, the Pesaran (39) CD test is used, and the conventional formulation is as follows:


(6) $$\begin{array}{r} {\text{CD}}_{Test}\;=\;\frac{\sqrt{2T}}{{\left[N\left(N-1\right)\right]}^{1/2}}\sum_{i=1}^{N-1}\sum_{k=1+i}^{N}T_{ik}.\;, \end{array}$$

The test under consideration depends on the independence of panel cross-sections in the selected emerging economies. In contrast, the alternative hypothesis will be accepted if the estimates are shown to be statistically significant at any of the one, five, or ten percent levels.

#### Unit Root

The current research uses a unit root estimator to address both SCH and CD issues using panel data. Specifically, this study uses the Pesaran (41) cross-sectional IPS (CIPS) test. A factor modeling description for cross-sectional dependency was presented by Pesaran (42). This strategy examines unexplained cross-sectional means. Pesaran (41) modifies ADF regression by including mean and first difference lag cross-sections. This approach generates cross-sectional dependence even if the panel is not balanced (*N*>*T* or *N*<*T*). The ADF cross-section could be expressed quantitatively as follows:


(7) $$\begin{array}{r} \Delta y_{i,t}=\theta_{i}+\beta_{i}^{*}y_{i,t-1}+d_{0}{\bar{y}}_{t-1}+d_{1}{\Delta \bar{y}}_{t}\;+\varepsilon_{it},,, \end{array}$$

where ${\bar{y}}_{t}$ is the mean of observations (N), and first lags of ${\bar{y}}_{t}$ and *y*_*it*_ maybe added to the said equation in order to deal with the serial correlation, given as:


(8) $$\begin{array}{l} \Delta y_{it}\;=\;\theta_{i}+\beta_{i}^{*}y_{i,t-1}+d_{0}\Delta {\bar{y}}_{t-1}+{\sum^{\text{​}}}_{j=0}^{n}d_{j+1}\Delta {\bar{y}}_{t-j}\\ \;+\;{\sum^{\text{​}}}_{k=1}^{n}c_{k}y_{i,t-k}+\;\varepsilon_{it},, \end{array}$$

To summarize, the CIPS (41) may be examined across the emerging economies by averaging the *t*-statistics for each cross-sectional unit (*CADFi*). The typical CIPS formulation is as follows:


(9) $$\begin{array}{r} CIPS=N^{-1}\;\sum_{i=1}^{N}CADF_{i},, \end{array}$$

The CIPS test assumed that a unit root is present in the time series (the null hypothesis).

#### Panel Cointegration Test

Since each variable satisfies the property of stationarity, therefore, it is crucial to analyze whether the long-run equilibrium nexus exists between the considered variables. In this concern, this study uses two-panel cointegration approaches, including the Westerlund (43) cointegration by demonstrating the variance ratio and the Pedroni (44) cointegration test. The latter test provides estimations for the Modified Phillips–Perron *t*, Phillips–Perron *t*, and Augmented Dickey–Fuller *t*. Concerning the propositions of these tests, both the tests asserted that there is no long-run cointegration between the variables. However, if the statistical values of these tests are significant at any of the 1, 5, or 10% levels, the null hypothesis may be rejected and the cointegration prevails between them.

#### Quantile Regression

Following the diagnostic tests (slope heterogeneity and cross-sectional dependence) and cointegration tests, we used the quantile regression approach devised by Koenker and Bassett (45) to investigate the long-run influence of the factors under consideration on MI. The motivation for using quantile regression is the non-normality issue or distribution of the data, meaning that typical estimation methods would not provide correct results. Furthermore, to avoid the overestimation and underestimation biases inherent in these typical approaches, this study employed the quantile regression technique, which provides the predicted coefficients at each specified quantile. Due to the fact that panel quantile regression allows for both individual and distributional variability, it provides precise insights into the relationship between the investigated variables (46). Moreover, quantile regression has a higher prediction performance than conventional regression, which only provides the average effect of explanatory variables on the dependent variable (47). Besides, the aforementioned estimator is advantageous owing to its treatment of the cross-sectional dependence and slope heterogeneity issues (48). Using Equations (12) and (13), the previously noted regression equations, i.e., Eqs. (1) and (2), could be turned into panel quantile regression forms below:


(10) $$\begin{array}{l} Q_{MI_{it}}(\theta \;\alpha_{i},\varphi_{t},X_{it})\;=\;\alpha_{i}+\varphi_{t}+\varphi_{1,\theta }GEF_{it}+\varphi_{2,\theta }EDU_{it}\\ \;+\;\varphi_{3,\theta }R\;\&D_{it}+\varphi_{4,\theta }GHE_{it}+\varepsilon_{it} \end{array}$$

(11) $$\begin{array}{l} Q_{MI_{it}}(\theta \;\alpha_{i},\varphi_{t},X_{it})\;=\;\alpha_{i}+\varphi_{t}+\varphi_{1,\theta }GEF_{it}+\varphi_{2,\theta }HC_{it}\\ \;+\;\varphi_{3,\theta }TI_{it}+\varphi_{4,\theta }GDP_{it}+\varepsilon_{it} \end{array}$$

From the equations above, the subscript θ demonstrates the quantile for each variable, which this study considers four, i.e., Q_25_, Q_50_, Q_75_, and Q_90_ in order to evaluate the influence of EDU, TI, GEF, HC, R&D, GHE, and GDP on MI in the selected emerging economies.

#### Panel Causality Test

The quantile regression technique yields estimated outcomes for each variable at a particular quantile but is limited in terms of displaying their causal connection. This research established causation using the Granger panel causality heterogeneity test developed by Dumitrescu and Hurlin (49). This test is more efficient and reliable in resolving the issue of the imbalanced panel (NT). In addition, it accounts for the cross-sectional dependency and variability of panel data (50).

## Results and Discussion

This segment deals with an explanation and a brief discussion of the results. Table 3 illustrates the descriptive statistics of the manuscript. Tables 4, 5 represent the outcomes of slope heterogeneity and cross-sectional dependence, respectively. Table 6 displays the findings of unit root testing. Table 7 shows the results of cointegration tests. Tables 8, 9 represent the quantile regression outcomes of both models (1 and 2) with particular graphical presentations. Table 10 demonstrates the panel causality test results of all study variables. Lastly, a fleeting discourse is held at the end of this section.

**Table 3:** Descriptive statistics and normality result.

	**MI**	**GEF**	**EDU**	**R&D**	**GHE**	**HC**	**GDP**	**TI**
Mean	0.196198	55.20448	24.31469	0.528679	2.257738	2.336298	27.91919	8.322054
Median	1.214860	55.94584	24.30404	0.614388	2.531189	2.352343	27.72514	8.262427
Maximum	3.121751	69.23077	26.23518	0.995142	4.422915	3.019475	30.23322	14.14756
Minimum	−12.19714	32.19804	21.57892	0.006234	0.549877	1.782071	26.68949	5.056246
Std. Dev.	2.969594	7.562053	0.952047	0.304223	1.173698	0.263703	0.854788	2.182289
Skewness	−1.798928	−0.505691	−0.462093	−0.239974	−0.074987	−0.022580	1.039707	1.018419
Kurtosis	6.544734	2.923728	3.371506	1.732799	1.403471	2.673765	3.584992	3.573222
Jarque-Bera	121.1711	4.886371	4.712653	8.721709	12.21414	0.515227	22.16436	21.26715
Probability	0.000000	0.086884	0.094768	0.012767	0.002227	0.772894	0.000015	0.000024
Observations	114	114	114	114	114	114	114	114

**Table 4:** Slope heterogeneity (38).

**Slope heterogeneity test**	**Statistics**
**Model-1**
~Δ	4.643***
~Δ^*Adjusted*^	5.613***
**Model-2**
~Δ	4.051***
~Δ^*Adjusted*^	4.897***

Significance level is denoted by *** for 1%, ** for 5%, and * for 10%.

**Table 5:** Cross-sectional dependence (39).

**Cross-section dependence**
**MI**	**GDP**
6.12***	16.39***
**GEF**	**GHE**
−1.42	7.40***
**HC**	**EDU**
14.29***	16.07***
**R&D**	**TI**
3.36***	16.05***

Significance level is denoted by *** for 1%, ** for 5%, and * for 10%.

**Table 6:** Unit root test (41).

**Variables**	**Intercept and Trend**	
	**I(0)**	**I(1)**
MI	−1.697	−3.854***
GDP	−1.532	−2.742*
GEF	−1.940	−4.187***
GHE	−1.814	−3.570***
HC	−2.780*	−3.349***
EDU	−2.836*	−3.816***
R&D	−1.916	−3.754***
TI	−1.526	−4.231***

Significance level is denoted by *** for 1%, ** for 5%, and * for 10%. I(0) is for level, and I(1) is for the first.

**Table 7:** Cointegration test.

**Pedroni Cointegration Test**
**Model-1**
Test	Statistics	*p*-value
Modified Phillips-Perron t	1.5289*	0.0631
Phillips-Perron t	−1.8778**	0.0302
Augmented Dickey-Fuller t	−1.7151**	0.0432
**Model-2**
Modified Phillips-Perron t	1.6715**	0.0473
Phillips-Perron t	−1.9190**	0.0275
Augmented Dickey-Fuller t	−1.9222**	0.0273
**Westerlund Cointegration Test**
**Model-1**
Variance ratio	−1.2849*	0.0994
**Model-2**
Variance ratio	−1.3978*	0.0811

Significance level is denoted by *** for 1%, ** for 5%, and * for 10%.

**Table 8:** Estimates of quantile regression Model 1.

**Variables**	**Quantiles**			
	**Q_**0.25**_**	**Q_**0.50**_**	**Q_**0.75**_**	**Q_**0.90**_**
GEF	−0.085* [0.048]	−0.058 [0.050]	−0.060*** [0.022]	−0.046*** [0.011]
EDU	0.394 [0.473]	0.263 [0.493]	−0.246 [0.219]	−0.403*** [0.114]
R&D	−3.937*** [0.883]	−0.366 [0.920]	1.540*** [0.409]	1.730*** [0.214]
GHE	−0.161 [0.309]	−0.387 [0.322]	−0.560*** [0.143]	−0.328*** [0.075]
*Constant*	−2.827 [10.975]	−0.993 [11.437]	11.295** [5.095]	14.204*** [2.662]

The dependent variable used here is MI. Significance level is denoted by ***, ** and * for 1, 5, and 10%. The standard error is provided in the brackets.

**Table 9:** Estimates of quantile regression Model 2.

**Variables**	**Quantiles**			
	**Q_**0.25**_**	**Q_**0.50**_**	**Q_**0.75**_**	**Q_**0.90**_**
GEF	−0.073* [0.042]	−0.069 [0.044]	−0.094*** [0.010]	−0.082*** [0.017]
HC	−8.824*** [1.407]	−5.676*** [1.468]	−3.203*** [0.354]	−3.231*** [0.566]
TI	−2.816*** [0.513]	−0.861 [0.535]	−0.482*** [0.129]	−0.524** [0.206]
GDP	5.732*** [1.344]	0.984 [1.402]	1.524*** [0.338]	1.558*** [0.540]
*Constant*	−113.063*** [32.099]	−2.723 [33.491]	−24.134*** [8.081]	−25.124*** [12.913]

The dependent variable used here is MI. Significance level is denoted by ***, ** and * for 1, 5, and 10%. The standard error is provided in the brackets.

**Table 10:** Dumitrescu–Hurlin panel causality.

***H***_**0**_	***Wald***_***Stats***_	** ${\bar{Z}}_{stats}$ **	**p−value**
GEF ⇏ MI	2.39840	−0.00133	0.9989
MI ⇏ GEF	6.17658***	3.14715	0.0016
EDU ⇏ MI	5.44364**	2.53637	0.0112
MI ⇏ EDU	2.50445	0.08704	0.9306
TI ⇏ MI	3.78234	1.15195	0.2493
MI ⇏ TI	2.20820	−0.15983	0.8730
HC ⇏ MI	6.58128***	3.48440	0.0005
MI ⇏ HC	114.059***	93.0495	0.0000
GHE ⇏ MI	6.37016***	3.30847	0.0009
MI ⇏ GHE	2.36875	−0.02604	0.9792
GDP ⇏ MI	4.05770	1.38142	0.1672
MI ⇏ GDP	2.87253	0.39377	0.6937
R&D ⇏ MI	2.65546	0.21288	0.8314
MI ⇏ R&D	0.89580	−1.25350	0.2100

Significance level is denoted by *** for 1%, ** for 5%, and * for 10%.

### Descriptive Statistics

The average values of all study variables are closely equal to their respective median values, demonstrating the balancing point of the information. The standard deviations denote how data are spread toward their mean values. Skewness and Kurtosis describe the normality and data precision. Byrne (35) demonstrated the range for both measures, i.e., between −2 and +2 for symmetrical distribution (skewness) but from −7 to +7 for peaked distribution (kurtosis). The statistical outcomes exemplify the precision and degree of tailedness in the distribution. Supplementarily, Jarque Bera validates the distribution. In general, the data are negatively skewed with the leptokurtic or heavy-tailed distribution.

### Slope Heterogeneity and Cross-Sectional Dependence

The left column of Table 4 signifies the statistics of slope heterogeneity by Pesaran and Yamagata (38) of both study models. The right column of Table 4 shows the statistics of the cross-sectional dependence (39) of all variables. Based on the existence of socioeconomical, financial, technical, etc. objectives in different countries across the panel or cross-section, there may prevail some similarities or dissimilarities that may give ineffective, biased, and inconsistent results. For efficient outcomes of the research, slope heterogeneity and cross-sectional dependence are employed in the process. The slope heterogeneity test is applied to determine the distance of slopes in cross sections individually. The statistical values in the table reject the null hypothesis of homogeneous coefficients at a 1% level of significance in both cases (Models 1 and 2). This suggests and validates that the coefficients of both models are heterogeneous in panel datasets across countries leading to groping the cross-sectional dependence of variables. The cross-sectional dependence examines the interdependence in the cross-section due to the presence of certain unobserved factors that indirectly impact in different ways. Pesaran's (39) test results show significant results with a 1% level of significance for all variables except government effectiveness. All variables are interdependent across the panel.

### Unit Root Testing

Based on the occurrence of cross-sectional dependence among the variables, the study primes toward the scrutinization of unit root, that is by transforming the traditional Augmented Dickey–Fuller regressions by cross-sectional averages (41) for panel root analysis at the level I(0) and the first difference I(1). The conventional ADF tests provide inefficient outcomes regarding cross-sectional averages. All eight variables except HC and EDU were insignificant at the level. The statistics represent substantial results with a 1% level of significance except for gross domestic product, which is significant at 10% at first difference. All variables rejected the null hypothesis at first difference. The negative coefficients reveal the robust existence of the unit root in the data values.

### Cointegration Tests

Since detecting Pesaran root results, each variable has a panel unit root that leads us to investigate the panel cointegration for examining the long-run (equilibrium) associations between the variables under study. Pedroni's panel cointegration tests consist of Modified Phillips–Perron (t), Phillips–Perron (t), and Augmented Dickey–Fuller (t), While the Westerlund cointegration test considers the variance ratio. The null hypothesis of these panel cointegration tests exhibits no cointegration, and it extends the unit root analysis to a multivariate method (approach). The statistics and p-values indicate the presence of panel cointegration by rejecting the null hypothesis at a 5% level of significance in Phillips–Perron t and Augmented Dickey–Fuller t tests in both models, whereas modified Phillips–Perron t is significant at 10% in Model 1 and 5% in Model 2. The Westerlund cointegration test has shown significant variance ratio results in both models (1 and 2) at a 10% level of significance. Thus, the criteria for rejecting the null hypothesis of no cointegration are satisfied.

In general, the results depict the existence of long-run associations of the variables. Gross domestic product, government effectiveness, government health expenditure, human capital, education, R&D, technological innovation, and malarial incidents have long-run relationships among them. In model 1, government effectiveness, education, R&D, and health expenses are associated with malarial incidents, while, in model 2, human capital, technological innovation, and GDP besides government effectiveness are linked to malarial incidents in the long run. The existence of a momentous effect of variables on malarial incidents, in the long run, is depicted in the outcomes. Hence, the long-run cointegration among the said variables in both models (1 and 2) is validated.

### Quantile Regressions

**Figure 2:**
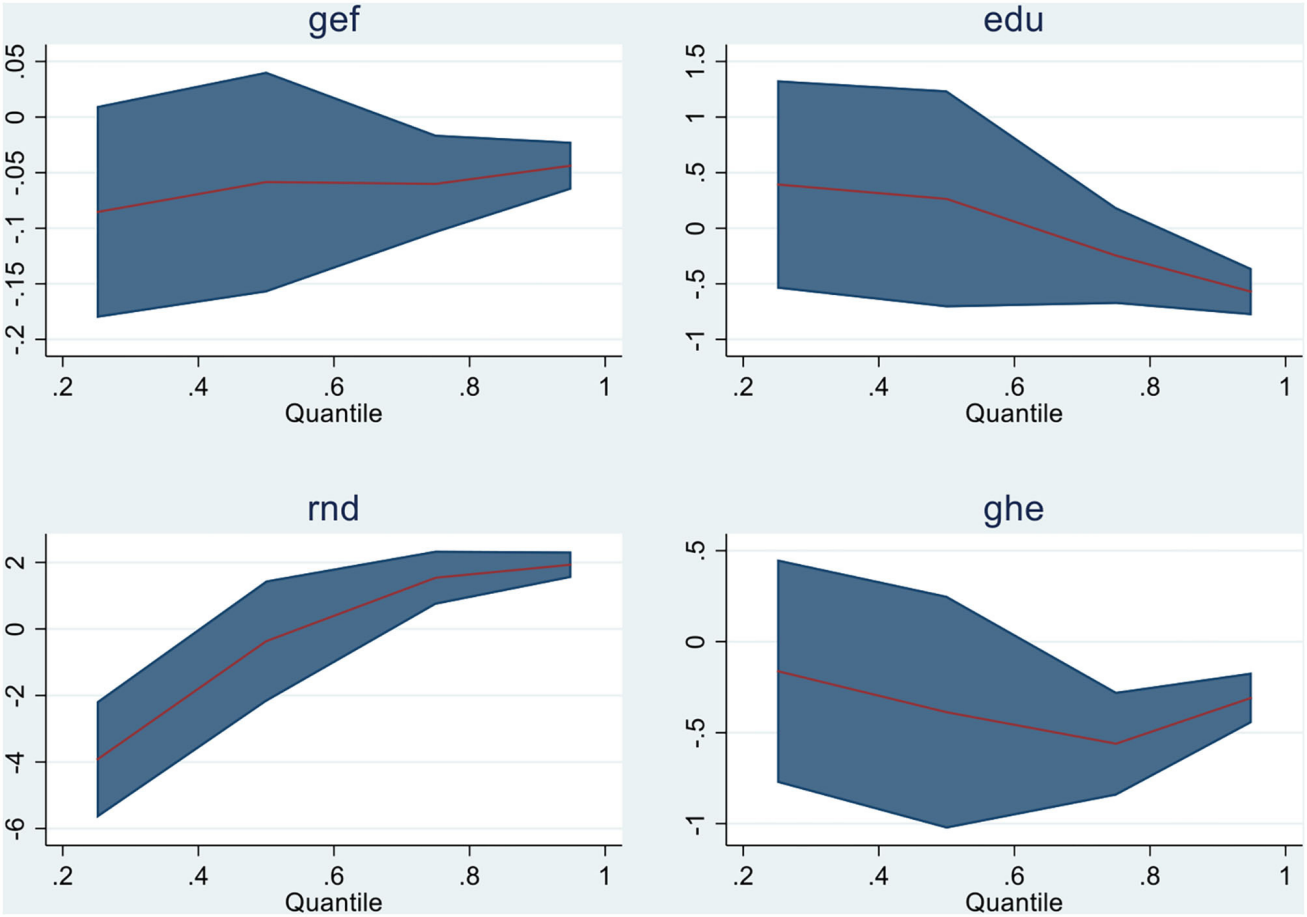
Graphical representation of quantiles for Model 1.

**Figure 3:**
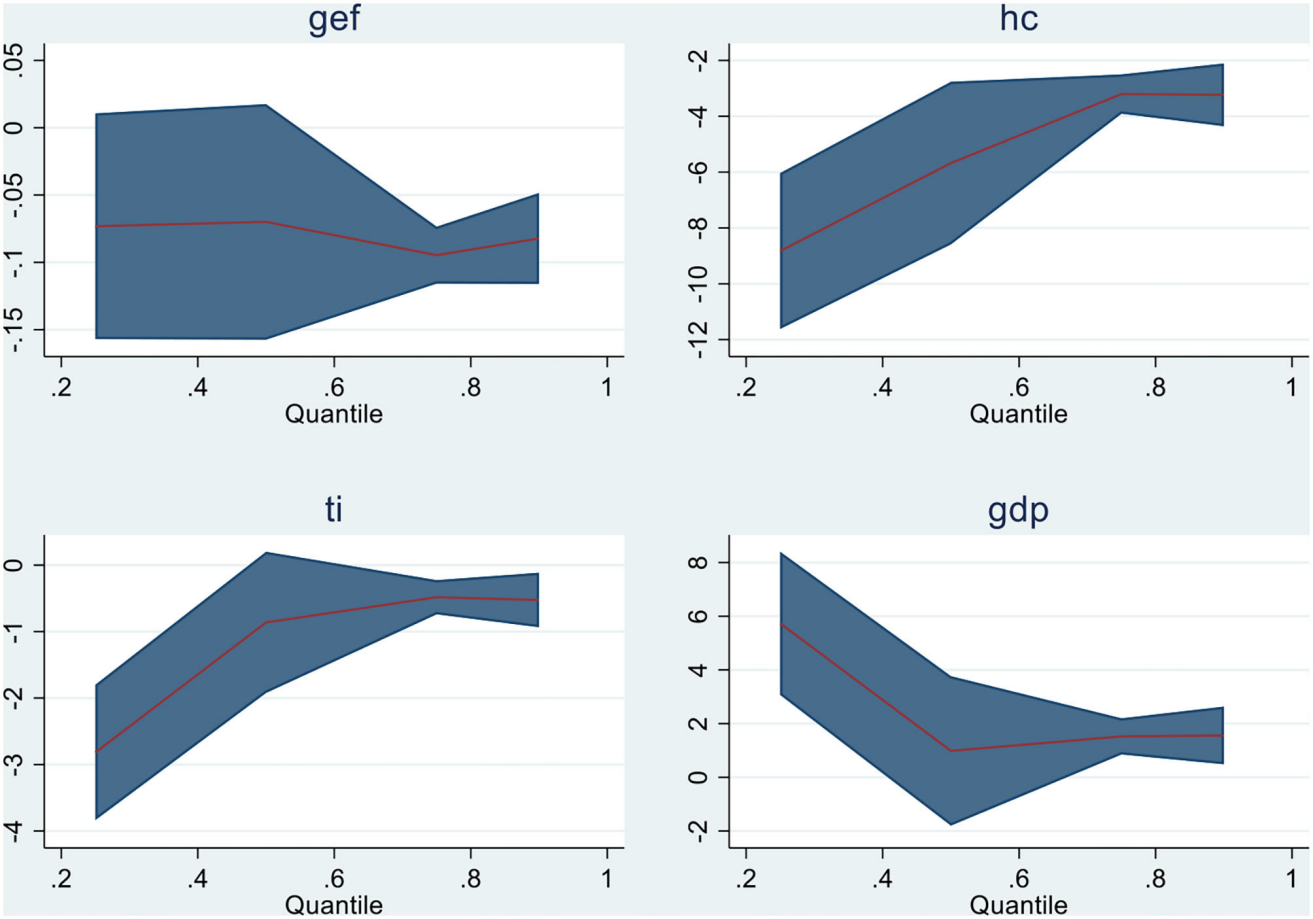
Graphical representation of quantiles for Model 2.

Quantile regressions are applied when linearity conditions are not fulfilled while the residual distribution is non-normal. Then, quantile regressions are utilized as extensions of standard ordinary least squares regressions. The findings of Quantile regressions for Model 1 are shown in Table 8 with graphical representation in Figure 2, and for Model 2, Table 9 and Figure 3 display the empirical findings and graphical presentation, respectively.

In model 1, all variables in Q (0.75) and Q (0.90) show substantial results except education in the 75th quantile. For illustration, GHE, EDU, and GEF show the negative relationship, that is, an increase in these variables negatively affects the malarial incidents in the emerging economies, while R&D has shown a strong negative association in Q (0.25) and Q (0.50) with malarial incidents. In model 2, all variables are negatively and significantly related to malarial incidents in almost all quantiles except gross domestic product, which is positively associated with the incidents of malaria in emerging countries (China, India, Brazil, Turkey, Mexico, and Indonesia). For illustration, advancement in technological innovation and human capital is inversely associated with malarial incidents, indicating a reduction in malarial incidents in E6 countries. Second, the increase in the economic growth of E6 countries increases the case of malaria in these countries,which is attributable to a positive relationship between the said variables. The graphical demonstration of panel quantiles of study variables is exhibited in Figures 2, 3.

### Panel Causality Test

Panel causality tests were applied to determine the interconnection of the considered study variables. Table 10 demonstrates the Dumitrescu–Hurlin causality results of all 14 sets of variable pairs. The panel causality depicts that only MI ⇏ GEF, EDU ⇏ MI, HC ⇏ MI, MI ⇏ HC, and GHE ⇏ MI pairs of variables have a causal and significant association at a 10% level of significance. These malarial incidence and human capital have bidirectional causality, that is, human capital and malarial incidents have a cause and effect relationship, whereas education and government health expenditures are unidirectionally associated with malarial incidents in the emerging six economies, although the remaining pairs (variables) have not shown any causal and significant associations by not rejecting the null hypothesis.

### Discussion Over Long-Run Empirics

Subsequently scrutinizing the descriptive stats and all variables' interdependence across the panel (initially), the overall empirical results of cointegration have been demonstrated in Table 8 (model 1) and Table 9 (model 2) with Figures 2, 3 and causality analysis is shown in Table 10 of the manuscript. The coefficient estimates of the emerging six countries depict an improvement in government effectiveness, education, government health expenses, human capital, and technological innovation and substantially support in decreasing the incidents of infectious disease (Malaria). The effect of these mentioned variables is found to be inversely linked to all malaria quantiles (nearly). The findings imply that E6 countries enhance their policies for healthcare to exterminate malaria. The findings are in line with the studies of Wei, Rahim, and Wang (10) and Omri et al. (7) regarding human capital, R& D, and health expenses with malarial incidents; government effectiveness and malarial incidents (11); and education awareness and malaria occurrence (12). However, theoretically, the study validates the suggestions of Huang et al. (30) for technological innovation for the eradication/prevention of disease incidents (malaria) in future. Upgrading in research and development for vaccines and medicines purposely reduces infectious diseases (1). Further, in the case of the emerging six economies, there is no substantial causal link between economic growth and malarial incidents. Understanding the causal association is essential in assessing the risk for infectious disease mortalities and gathering more evidence for further research and development for disease handling. With more population, the disease burden grows. However, with effective implementation of healthcare, it can be cured permanently. Together with technology, effective governmental policies, R&D, and informative awareness can mitigate mortalities and disease spread.

## Conclusion and Implications

The current study aims to examine the effectiveness of government (GEF), education (EDU), research and development (R&D), and government health expenditure (GHE) over the malarial incidents in the first model. Whereas in the second model, the influence of human capital (HC), technological innovation (TI), and gross domestic product (GDP), besides government effectiveness (GEF) over malarial incidents, are analyzed. The scarceness of studies in the standing literature concerning the association between malarial incidences with the abovementioned factors leads to assessing the said connection. Therefore, the motivation of the study is to evaluate the consequence of a varied range of variables on incidents of malaria, which is still a crucial issue that is affecting the world economically, socially, and individually the health of people, causing millions of deaths per year. The study covers the debate on malarial infection incidents in the case of E6 nations, which is innovative research in the speculative and empirical literature.

The causality test demonstrates that malarial incidence and human capital have a bidirectional causal association, whereas education and government health expenditures are unidirectionally linked to malarial incidents in the emerging six economies. Otherwise, the remaining variables have no cause and effect associations. Therefore, the empirical results are consistent with some studies concerning different variables' influence on infectious disease (malaria). As *per se*, regarding the association of human capital, research and development, and government health expenditures with malarial incidents, the present study is in line with the studies of Wei et al. (10) and Omri et al. (7); for the effectiveness of government with malarial incidents, Liang et al. (11) and Sarpong and Bein (19) are reliable with the findings; and also, the educational awareness with malaria occurrence (12) is consistent. These declared empirical pieces of evidence from existing literature are in line with the outcomes in one way or another. However, from the theoretical perspective, the study validates the suggestions of Huang et al. (30) for technological innovation for the eradication/prevention of malarial incidents. No causal association but the positive impact of GDP with malarial incidents was observed, which is a novel contribution to the literature.

Meanwhile, the emerging countries are likely to expand in the coming future as global nations. Yet, these emerging economies such as Indonesia, India, Brazil, and Mexico have reported millions of cases of malaria and deaths per year. Therefore, the eradication of malaria and other infectious diseases must be their priority with other economical factors for sustainable growth. Globally, malaria has been a prime health concern for decades. It is essential to find the infected number of cases or incidents because the occurrence and incubation time are different and entirely reliant on the strain of parasite (Plasmodium vivax), infection type, etc. In addition, the number of incidents may increase or decrease depending on a certain number of factors that are important to identify and lessen the malaria burden across communities, countries, and at a regional level.

The precise findings of panel estimations revealed substantial implications. The comprehensive outcomes depict that health expenditure is not the only sustainable solution for malaria control. The role of technological innovation in disease diagnostic, medicinal advancement, and treatment plays a noteworthy part. With innovative research and development methods for vaccination, development of a permanent cure is necessary. Also, important research is needed for the recognition of human samples instead of an animal because animal representations do not certainly reflect the human scenario. Malaria is an ancient life-threatening disease that reemerges after a certain period; therefore, a permanent treatment and drug discovery are required for the prohibition with the help of product and technological innovation. Educational programs for good hygiene and clean environment aid in maintaining sustainable development goals. Efforts for malaria control and basic preventive measures (use of spray insecticides, bed nets, and repellents) with the help of educational awareness for knowledge of malaria can be fruitful. The national governments must create those awareness programs together with sustainable health plans for malaria prevention. Besides, effective immediate control will also reduce mortalities and morbidities in the country. Further, government and NGOs provide basic free facilities and services together with subsidies for disease prevention/control. Healthy human capital is effective in healthcare to provide services for the betterment of society and the economy because a well (healthy) individual can work at full capacity. Therefore, health services must be provided at domestic and global levels. By implementing preventive policies as adopted by other developed countries such as the United States in the 50s by utilizing insecticides and drainage ditches, etc., China has worked to vanquish malaria. They implemented robust technologies for malaria control and updated the health programs to firm incorporation of the disease management.

The limitation of the research can be extended for forthcoming exploration purposes. Foremost, the study is restricted to the emerging six economies with the inclusion of various variables for the first time. Therefore, it can be scrutinized in the future in tropical and poor economies considering the present study variables. For this very reason, poor economies are more vulnerable to malaria and other infectious diseases, so this can be investigated in those countries with the same or new specifications. Second, the impact and linkage of other infectious diseases, endemics, or pandemics with the study variables can be examined in the future in E6 nations or other sample countries that would be resourceful in health and economic policymaking and creating strategic awareness programs, because the researcher needs to gather more evidence on malarial incidents or cases to scrutinize and assess the risk for malaria morbidity and mortalities. In addition, the research can be extended on the economic impact of treatment drugs and vaccines on public health.

## Data Availability Statement

The original contributions presented in the study are included in the article/supplementary material, further inquiries can be directed to the corresponding author.

## Author Contributions

The author confirms being the sole contributor of this work and has approved it for publication.

## Conflict of Interest

The author declares that the research was conducted in the absence of any commercial or financial relationships that could be construed as a potential conflict of interest.

## Publisher's Note

All claims expressed in this article are solely those of the authors and do not necessarily represent those of their affiliated organizations, or those of the publisher, the editors and the reviewers. Any product that may be evaluated in this article, or claim that may be made by its manufacturer, is not guaranteed or endorsed by the publisher.

## Footnotes

^2^ https://www.unicef.org/health/childhood-diseases#:~:text=prevention%20and%20treatment.-,Malaria,cent%20of%20global%20malaria%20deaths

^3^ https://www.google.com/search?q=Number+of+malaria+cases+per+year+in+Indonesia,+India,+Brazil,+and+Mexico+Source:+Statista+2022.&sxsrf=ALiCzsb7YlOIDly-YKoo2HGvOBVD9UE6Aw:1655928090053&source=lnms&tbm=isch&sa=X&ved=2ahUKEwjI1dLs7MH4AhXHhv0HHUcDCiwQ_AUoAnoECBoQBA&biw=1366&bih=617&dpr=1
